# Prospects for Narrow Band Imaging Magnification Endoscopy in Oral Lesions: Recommendations from Oral and Maxillofacial Surgeons and a Gastroenterologist

**DOI:** 10.3390/cancers17010021

**Published:** 2024-12-25

**Authors:** Toshimi Chiba, Airi Ota, Taifu Hirano, Tadashi Kawai, Atsushi Ogawa, Hiroyuki Yamada

**Affiliations:** 1 Division of Internal Medicine, Department of Oral Medicine, School of Dentistry, Iwate Medical University, Morioka 020-8505, Iwate, Japan; 2 Division of Oral and Maxillofacial Surgery, Department of Oral and Maxillofacial Reconstructive Surgery School of Dentistry, Iwate Medical University, Morioka 020-8505, Iwate, Japan; ootaa@iwate-med.ac.jp (A.O.); thirano@iwate-med.ac.jp (T.H.); kawait@iwate-med.ac.jp (T.K.); atsushio@iwate-med.ac.jp (A.O.); yamadah@iwate-med.ac.jp (H.Y.)

**Keywords:** narrow band imaging, magnification endoscopy, intraepithelial papillary capillary loops, oral potentially malignant disorders, oral squamous cell carcinoma

## Abstract

Narrow band imaging (NBI) magnification endoscopy for the diagnosis of early-stage oral cavity-related cancer and precancerous lesions can recognize oral lesions as brownish areas, and can observe intraepithelial papillary capillary loops (IPCLs) in the mucosa and submucosa to make a qualitative diagnosis of the lesion and highlight the mucosal surface microstructure to facilitate appropriate diagnosis and early treatment. IPCLs are classified from Type 0 to IV: Type 0 is normal mucosa or no blood vessels observed, e.g., in keratinization; Type I is mainly normal mucosa; Type II is mainly inflammatory sites or non-malignant lesions; Type III is mainly precancerous or suspected malignant lesions; and Type IV is cancerous or malignant lesions. NBI magnification endoscopy is a useful noninvasive method for identifying the malignant transformation of oral potentially malignant disorders (OPMDs). Oral lesions classified as IPCL Type II or higher are atypical epithelial or oral squamous cell carcinoma (OSCC); oral biopsy is recommended for early and accurate diagnosis, and is an indicator of the appropriate biopsy site in the follow-up for OPMDs. In the future, the accuracy of NBI magnification endoscopy for malignant transformation of OPMDs and OSCC will be further confirmed.

## Simple Summary

This review explores the interpretation of intraepithelial papillary capillary loop (IPCL) classification in the narrow band imaging (NBI) magnification endoscopy of oral lesions. IPCLs are classified from Type 0 to IV. NBI magnification endoscopy is a useful noninvasive method for identifying the malignant transformation of oral potentially malignant disorders (OPMDs). Oral lesions classified as IPCL Type II or higher are atypical epithelial or oral squamous cell carcinoma (OSCC); oral biopsy is recommended for early and accurate diagnosis, and is an indicator of the appropriate biopsy site in the follow-up for OPMDs. The accuracy of NBI magnification endoscopy regarding the malignant transformation of OPMDs and OSCC will be further confirmed in the future. It is expected that NBI magnification endoscopy will be widely used by dentists in hospitals and general dental clinics.

## 1. Introduction

The diagnosis of benign or malignant oral lesions and of malignancy in cancer or precancerous lesions relies on histopathological examination. In addition, early-stage oral squamous cell carcinoma (OSCC) has a good survival rate and quality of life (QOL) after treatment. Early diagnosis and treatment of lesions is desirable to improve the treatment outcome and prognosis of OSCC. For the diagnosis of early-stage oral cavity-related cancer and precancerous lesions, examination of lesions using an endoscope in addition to conventional macroscopic examination enables observation of lesions in close proximity and frontal observation of lesions in areas that cannot be observed with a macroscopic examination, which can lead to clearer decisions regarding treatment strategy.

The magnifying endoscope function has been used since the 1990s. Currently, in endoscopic observation of oral lesions under special light, autofluorescence imaging (AFI) displays cancerous lesions as reddish purple with weak autofluorescence and normal mucosa as green with strong autofluorescence, confirming the presence of the lesion [1]. Narrow band imaging (NBI), an optical image enhancement technique, is an endoscopic examination using special light, and in combination with magnification, enables detailed observation of dilated and irregular capillary vessels in the mucosa and submucosa for qualitative diagnosis of lesions and also highlights microstructures on the mucosal surface. NBI is characterized by its ability to diagnose malignant or atypical lesions using magnification of about 100x in addition to lesion detection. AFI is effective in diagnosing the presence of a lesion, while NBI is useful in diagnosing intraepithelial papillary capillary loop (IPCL) patterns by magnifying the lesion, and for qualitative diagnosis of the lesion. This enables appropriate diagnosis and early treatment, prevents progression to advanced cancer, and contributes to improved prognosis. In the future, NBI magnification endoscopy is also expected to be applied to endoscopic treatment of OPMDs and early-stage OSCC. Furthermore, attempts have been reported to perform pathological diagnosis of oral mucosal lesions in real time using endocytoscopy, a contact-type light microscopy system with 380x magnification, to observe both structural and cytological atypia [2].

This article summarizes the selection of endoscopic instruments, observation methods, and endoscopic findings and their interpretations for NBI magnification of oral lesions, and provides useful information for actual clinical practice.

## 2. Search Strategy for OSCC or OPMDs and NBI

The bibliography, available on PubMed and Web of Science databases (up to 1 June 2024), was selected using the following keywords: “Oral squamous cell carcinoma”, or “OSCC”, or “Oral potentially malignant disorders”, or “OPMDs”, and “NBI” or “Narrow Band Imaging”. The inclusion criteria were studies about NBI use for OSCC or OPMD samples and the classification of NBI patterns based on IPCL grading criteria. Appropriate literatures were analyzed from references mentioned in previously selected articles. English was the language used. The exclusion criteria were animal models, case studies, and studies using optical evaluation criteria other than IPCLs.

## 3. Principles of NBI

NBI is an image enhancement function that uses two wavelengths, 415 nm and 540 nm, narrowed by a dedicated optical filter. As the peak absorption region of oxidized hemoglobin in blood is 415 nm and 540 nm, and short wavelengths such as 415 nm and 540 nm are scattered in the superficial layers of biological tissues, NBI has an image enhancement function that improves the visibility of surface microstructure and microvasculature under an endoscope in combination with a magnifying endoscopy.

(1) Non-magnification (diagnosis of the presence of a tumor)

The effectiveness of NBI in non-magnification observation has been demonstrated in the pharyngeal and esophageal region with a high level of evidence. Superficially limited cancer of the head and neck and esophageal carcinoma (multiple superficial carcinomas in the esophagus) can be detected with a significantly higher detection rate and diagnostic accuracy by NBI than by white-light imaging [3,4].

(2) Observation with magnification (qualitative and quantitative diagnosis of tumors)

NBI is highly effective for the qualitative and quantitative diagnosis of esophageal squamous cell carcinoma, gastric cancer, and colorectal tumors because it enables observation of the microvasculature and surface microstructure when used in combination with magnifying endoscopy. In the esophagus, observation of intraepithelial papillary capillary loop (IPCL) atypia by a combination of NBI and magnification is effective not only for the diagnosis of cancerous or non-cancerous lesions, but also for the diagnosis of the extent and depth of the lesion [3,4].

## 4. Current Status of NBI Magnification Endoscopic Observation

NBI is a method that uses a special imaging technique to visualize mucosal and submucosal IPCLs by magnifying the lesion and making it easier to identify early-stage cancers compared to conventional observation [3]. According to a systematic review, NBI, along with white-light imaging (WLI), is an important method for improving the diagnostic accuracy of head and neck cancer [4,5]. Furthermore, NBI magnification is characterized by its ability to diagnose the atypia or grade of malignancy of the lesion in addition to the presence of the lesion. There are cases in which clinically malignant lesions are suspected, but biopsy tissue shows only inflammatory findings and no atypical or malignant cells are detected, resulting in cases being followed up. As biopsy is based on a portion of the lesion and does not necessarily reflect the entire lesion, when NBI magnification endoscopy reveals Type III or higher findings, biopsy should be performed on suspicion of severe atypia; when Type II findings are present and biopsy results show only inflammatory findings, the patient should be followed up for a certain period of time. If a tumor is suspected clinically, it is useful for both the patient and the clinician to perform a complete resection of the tumor in the early stage of the disease and to perform a histological examination of the entire tumor as a diagnostic treatment to confirm the diagnosis. In other words, this is the concept of early case detection and early treatment.

In esophageal carcinoma, a squamous cell carcinoma of the same histological type as oral cancer, upper gastrointestinal endoscopy using NBI has been already performed in practice. In addition to detecting neoplastic lesions, their grades are diagnosed by NBI magnifying endoscopy, and endoscopic treatment may be performed without biopsy if superficially limited esophageal cancer is suspected. This is because biopsy scars can make endoscopic treatment difficult. Oral lesions are also highly malignant squamous cell carcinomas, and the prognosis is not good; it is important for dentists who treat oral malignant lesions to further promote NBI magnification endoscopy and to accumulate and review cases. Early detection and early treatment of malignant oral lesions can minimize the invasiveness of surgery on these lesions and greatly improve the prognosis.

## 5. Examination Procedures and Observation Methods

Regarding indications and contraindications for the use of oral endoscopy, there are no restrictions to its use because diagnoses are mainly made by intraoral observation. In patients taking anticoagulants or antiplatelet drugs or with a bleeding tendency, some bleeding may occur when the endoscope comes into contact with the oral mucosa, but this is not a major problem and hemostasis is considered possible without invasive endoscopic procedures such as biopsy, although careful handling is required.

(1) Pre-treatment

No dietary restrictions are required one day before the examination, and food intake on the day of the examination is not a problem if only oral observation is performed. Oral rinsing should be performed prior to the examination. Antispasmodics, sedatives, and analgesics are considered unnecessary. If oral mycosis is found, the mucosal lesion should be observed after treatment with antifungal agents because the white coat of infection makes it difficult to observe the IPCL. Although elimination of white coat on the oral mucosa is possible by gauze wiping, preexisting inflammation may modify the IPCL of the lesion.

(2) Endoscopic instruments and observation methods

The endoscopic video scope systems EVIS LUCERA ELITE, CV-290 (EVIS LUCERA ELITE Video System Center), CLV-290 (EVIS LUCERA ELITE High Intensity Light Source), and GIF-H290Z upper gastrointestinal endoscope (Olympus Corporation) are used. Lesions can be observed with macroscopic examination and normal light (white light), switched to NBI to observe the brownish area, and magnified (up to 85x) to observe the mucosal and submucosal IPCL of the lesion and the microstructure of the mucosal surface in detail.

Specifically, a lesion is observed and photographed using white light to confirm the degree of erythema, surface irregularity, and the extent of the lesion in the area of abnormal findings in the macroscopic examination. Next, the lesion is observed and photographed using the same procedure as for white light by switching to NBI. The lesion’s center, margins, and vascular structures (IPCLs) at the border between the lesion and the normal mucosa are observed and photographed using NBI magnification endoscopy. As the magnification function of the GIF-H290Z is capable of up to 85x, it should be used as appropriate for observation. It is also recommended to observe the lesion with a cap, but be careful of bleeding due to contact between the endoscope and the lesion. Iodine staining should be performed to confirm the lesion. Biopsy should be performed after endoscopic observation.

## 6. NBI Magnification Endoscopy of Oral Lesions

The classification of the morphology of microvascular loops in the oral mucosa by NBI magnification endoscopy and its correlation with the atypia of lesions has been reported [6,7,8]. NBI magnification endoscopy has been shown to be effective in detecting atypia/malignant lesions in the oral cavity and pharyngolarynx [9,10,11]. NBI magnification endoscopy in the oral cavity has been reported to have higher sensitivity, specificity, positive predictive value, negative predictive value, and accuracy in the diagnosis of OSCC compared to WLI, and is useful for the recognition and accurate evaluation of neoplastic lesions in the oral cavity [9,12].

(1) IPCL classification by NBI magnification endoscopy [6,7,8]

IPCLs are classified from Types 0 to IV.

**Figure 1:**
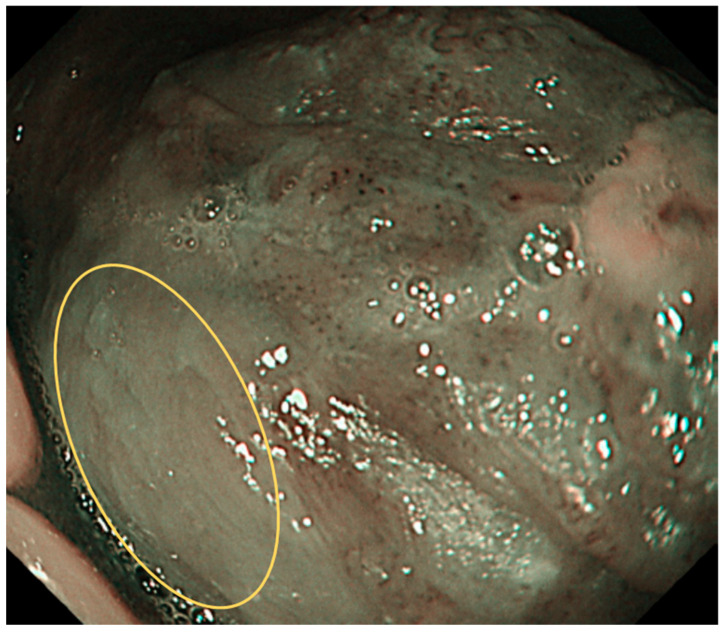
Type 0 is shown in the circle: No vessels are observed due to normal mucosa or keratinisation, etc.

Type 0: No vessels are observed due to normal mucosa or keratinization (Figure 1).

**Figure 2:**
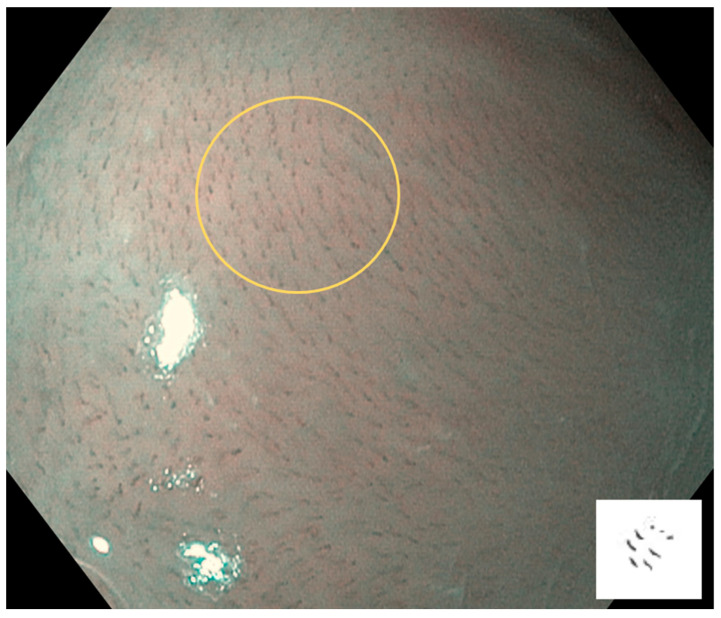
Type I is shown in the circle: Regular brown dots are observed when IPCLs are perpendicular to the mucosa, and waved lines are observed when running parallel. Most of the mucosa is normal.

Type I: Regular brown dots are observed when IPCLs are perpendicular to the mucosa, and waved lines are observed when running parallel. Most of the mucosa is normal (Figure 2).

**Figure 3:**
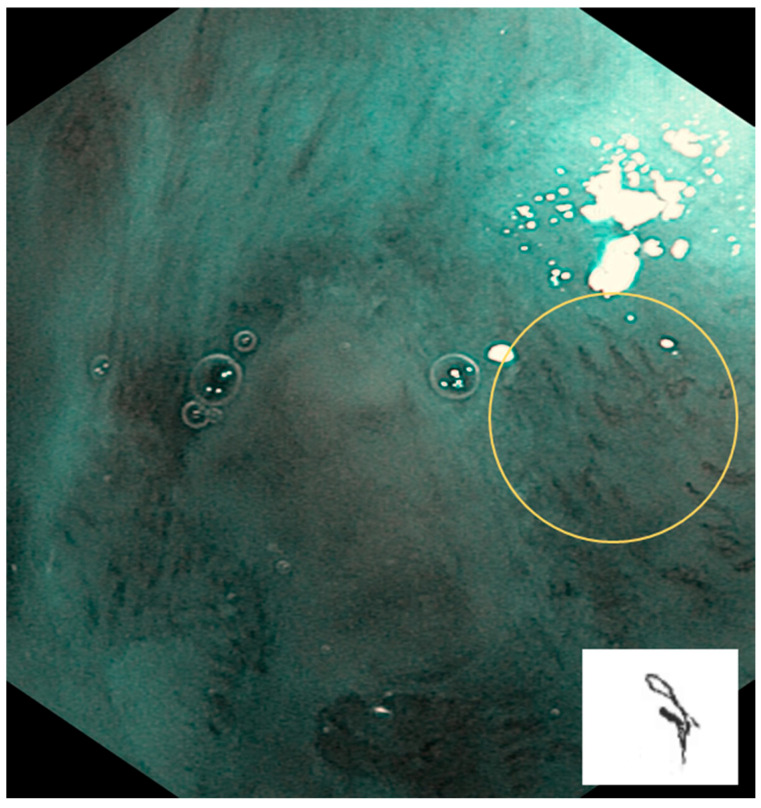
Type II is shown in the circle: IPCLs are dilated and crossing. They are mainly at inflammatory sites or non-malignant lesions.

Type II: IPCLs are dilated and crossing. They are mainly at inflammatory sites or non-malignant lesions (Figure 3).

**Figure 4:**
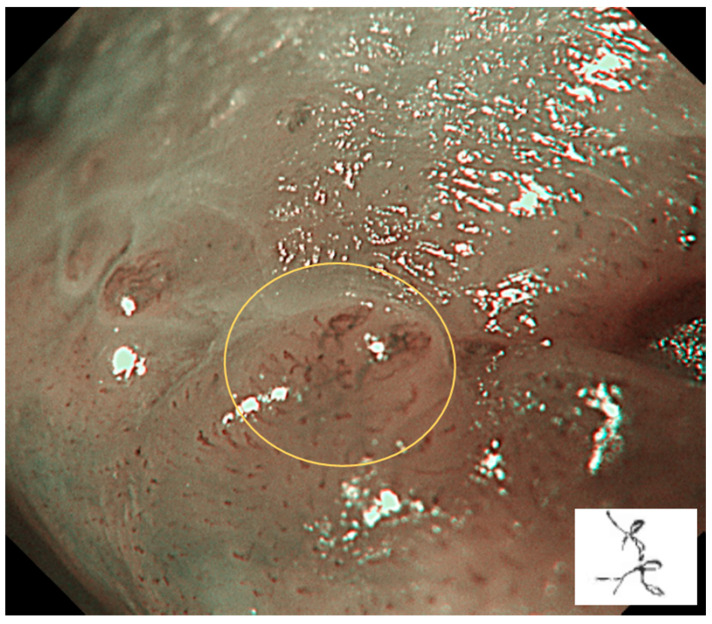
Type III is shown in the circle: IPCLs are further elongated and meandering. This is mainly seen in precancerous or suspected malignant lesions.

Type III: IPCLs are further elongated and meandering. This is mainly seen in precancerous or suspected malignant lesions (Figure 4).

**Figure 5:**
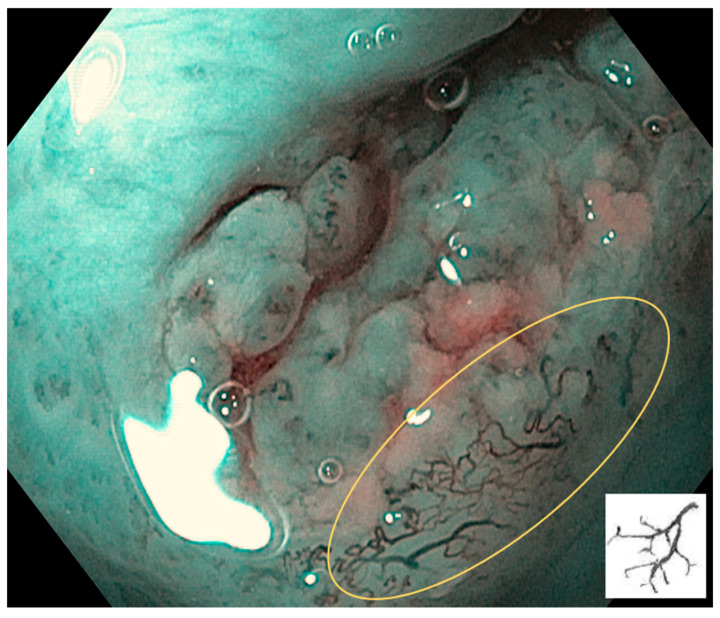
Type IV is shown in the circle: IPCLs are characterized by large vessels, destruction of looped vascular structures, and angiogenesis. This suggests the possibility of cancerous or malignant lesions.

Type IV: IPCLs are characterized by large vessels, destruction of looped vascular structures, and angiogenesis. This suggests the possibility of cancerous or malignant lesions (Figure 5).

(2) Treatment of IPCL classifications

It has been reported that 97.7% of IPCL Types 0, I, and II were classified as non-neoplastic lesions (inflammation, etc.), while 78.9% of IPCL Types III and IV were neoplastic lesions [13]. Furthermore, as IPCL Type IV is significantly associated with OSSC, when using NBI magnification endoscopy, the detection of IPCL Type IV in the follow-up for oral lesions may indicate the presence of OSSC [12].

## 7. Interpretation of IPCLs

Oral potentially malignant disorders (OPMDs), such as erythroplakia, erythromatous leukoplakia, leukoplakia, oral submucous fibrosis, and dyskeratosis congenita, are lesions or conditions that can lead to cancer. NBI magnification has been used for better assessment of OPMDs, identification of oral and oropharyngeal squamous cell carcinoma (SCC), and diagnosis of surgical margins of head and neck malignancies. NBI magnification has shown great potential to improve the detection rate of OPMDs, facilitate the evaluation of oral and oropharyngeal SCC, and lead to early detection of recurrent OSCC. Although further studies are needed to correlate IPCLs with clinical, histopathological, and molecular biological parameters, evidence is being proposed for a new gold standard of endoscopic evaluation in oral and head and neck oncology [14,15].

In oral lichen planus (OLP), the detection of IPCL Types III and IV on NBI is considered useful in the follow-up of the malignant transformation of lesions [16], and diffuse and severe chronic inflammation of OLP generally causes an abnormal vascular pattern [17]. In oral leukoplakia, when a high-grade IPCL is seen on NBI magnification, the risk of OSCC is high, and proliferative verrucous leukoplakia tends to be malignant, suggesting that tumor angiogenesis preceding epithelial malignant transformation may be observed on NBI magnification [18].

When the diagnostic accuracy of IPCLs in detecting OPMDs was compared to those of conventional visual inspection (CVI), WLI, and NBI, in OLP and leukoplakia a wide range of lesions could be identified using WLI or NBI compared to CVI, and NBI could detect lesions in larger areas compared to WLI. IPCLs are Types 0–II in 75.0% of OLP, various IPCLs are seen in leukemia, and all cases of OSCC have Type III or IV IPCLs [19]. The diagnostic accuracy of OSCC was 100% sensitivity and 80.9% specificity when a severe IPCL was observed. The endoscopic observation of oral mucosal lesions is also useful to determine the margins of these lesions, and NBI magnification endoscopy of an IPCL can be an important examination for the early detection of OSCC in OPMDs lesions [19,20].

According to a recent systematic review, NBI magnification is an effective noninvasive method for identifying the malignant transformation of OPMDs, and as detailed observation of lesions may reveal the presence of neoplastic lesions, biopsy of oral lesions classified as IPCL Type II or higher is recommended for early and accurate diagnosis of atypical epithelium or OSCC [15].

## 8. Limitations of NBI Magnification Endoscopy

Although NBI has proven to be an efficient diagnostic tool, NBI is inaccurate in thick keratinized mucosa because of the inability to observe blood vessels. A study using the vascular pattern around the lesion as an evaluation index showed that approximately 28% of thick, homogeneous vitiligo with IPCL Type I surrounding the lesion was actually accompanied by atypia [21]. In addition, the presence of hemorrhage in the NBI field of view made it difficult to observe the IPCL [22], and the ulcer surface of an oral ulcer lesion obscured visualization of the IPCL. In normal mucosa, an IPCL is difficult to observe on the dorsum of the tongue, hard palate, and gingiva, etc. Note that OLPs are commonly observed on the buccal mucosa and lower lip and are only recognized as lesions in areas where they can be visualized [7,19,23]. The keratinized layer and pigmentation make the observation of an IPCL impossible. The presence of a keratinized layer is one of the limitations of this observation. And the effects of the use of keratolytic agents on IPCLs have not been well understood. Therefore, pre-treatment of IPCLs for NBI magnification endoscopic observations requires further study. Although the cost of installing endoscopic equipment is necessary to some extent, the cost associated with the observation technique is almost negligible. Early detection of lesions by NBI magnification endoscopic observation and early treatment may lead to lower treatment costs and improved prognosis.

## 9. Future Prospects

On WLI endoscopy, lesions of irregular surfaces and indistinct margins were observed in 56% of OLPs with keratinized white lesions in a background of redness of mucosa, and lesions of irregular surfaces of OSCC were observed in 92% with nodular or verrucous white lesions in a background of redness of mucosa. On the other hand, 84% of leukoplakia have smooth surfaces and circumscribed lesions. Therefore, the appearance of an irregular surface in leukoplakia suggests the occurrence of OSCC. Details of the association between surface irregularities and IPCLs are not well known, but IPCL Type III is present in 25% of OLPs, which might reflect severe atypical lesions or OSCC. Lesion sites of IPCL Type III or Type IV indicate the possible presence of malignancy, and therefore provide an indicator of the appropriate biopsy site and minimize multiple biopsies [24]. For OPMDs, a strict diagnosis of IPCL and regular follow-up with NBI magnification endoscopy is necessary. It has been reported that lesions should be reevaluated by NBI one month after screening [24].

## 10. Conclusions

The IPCL classification of OPMDs and OSCC with NBI magnification endoscopy is the optimal approach for the early detection of the malignant transformation of OPMDs. NBI magnification endoscopy is a relatively accurate and effective noninvasive diagnostic tool, and biopsy is strongly recommended for lesions diagnosed as IPCL Type II or higher. Further case studies are needed to confirm the accuracy of NBI magnification endoscopy for oral diseases.

## Data Availability

Not applicable.
